# Testicular Adrenal Rest Tumors in a Patient With Congenital Adrenal Hyperplasia

**DOI:** 10.7759/cureus.28350

**Published:** 2022-08-24

**Authors:** Su-Yuan Yu, Kelly M Freed

**Affiliations:** 1 Department of Radiology, University of South Florida (USF) Morsani College of Medicine - Lehigh Valley, Allentown, USA

**Keywords:** testicular ultrasound, 17-hydroxyprogesterone, adrenocorticotropic hormone, bilateral testicular tumors, congenital adrenal hyperplasia, testicular adrenal rest tumors

## Abstract

Ultrasound is the imaging examination of choice for evaluation of suspected testicular pathology. The differential diagnosis of bilateral testicular lesions includes malignancy such as lymphoma and metastases, infection, and, uncommonly, adrenal rest tumors. We present a patient who developed bilateral testicular adrenal rest tumors after years of poorly controlled congenital adrenal hyperplasia, possibly due to chronically elevated adrenocorticotropic hormone stimulating the growth of testicular stem cells. Our patient also has a testicular ultrasound appearance that is hyperechogenic, rather than hypoechogenic as commonly described in the literature. Treatment adherence is important in the management of congenital adrenal hyperplasia, as testicular adrenal rest tumors may eventually lead to infertility.

## Introduction

Testicular adrenal rest tumors (TARTs) are rare tumors associated with congenital adrenal hyperplasia (CAH). CAH is an autosomal recessive disorder of the adrenal gland characterized by an enzymatic defect in the adrenal steroid hormone production pathway, leading to low cortisol and aldosterone levels. The lack of negative feedback from cortisol causes the pituitary gland to release an increased amount of adrenocorticotropic hormone (ACTH), resulting in adrenal hyperplasia. While the incidence is between one to two in 20,000, the estimated prevalence of TARTs in male patients with CAH is 37% [1,2]. Prevalence increases significantly after onset of puberty [3]. Although benign, these lesions are the leading cause of infertility in male patients with CAH. In this report, we review a case of TART with an atypical ultrasound appearance and the relevant literature.

## Case presentation

A 36-year-old male presented to an outpatient endocrinology office for a regular follow-up visit for CAH. The patient was diagnosed in infancy and had a concurrent mineralocorticoid deficiency. At presentation, he was asymptomatic other than having an unplanned weight loss of 10 pounds following a coronavirus disease 2019 (COVID-19) infection. The patient was mildly hypertensive (blood pressure 148/85 mmHg) and a physical exam revealed firm testes, with nodular texture, particularly of the right testis. In addition, he had hyperpigmentation of his skin and gums.

The patient has been placed on long-term glucocorticoid replacement therapy with hydrocortisone, fludrocortisone, and dexamethasone and was followed by an endocrinologist. For several years, the patient was poorly compliant with this medication regimen due to the loss of his health insurance. He was hospitalized once for adrenal crisis during this period.

**Figure 1:**
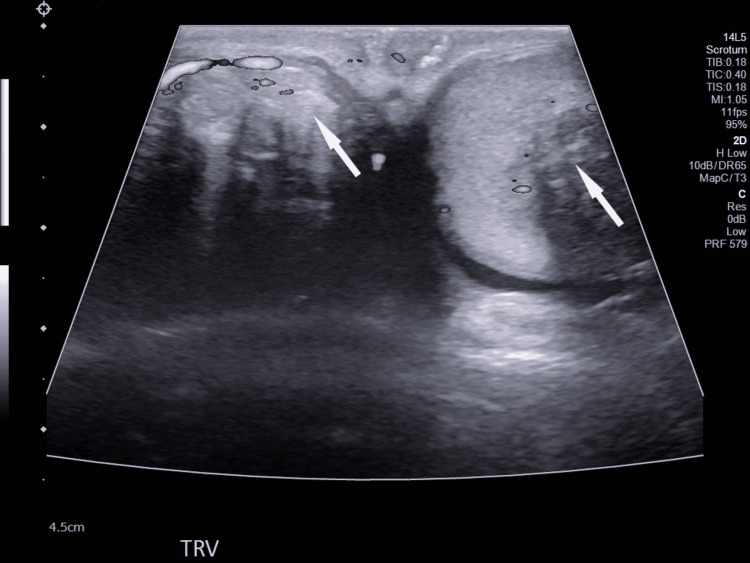
Color duplex transverse ultrasound image of the bilateral testicles demonstrating multiple, heterogeneous, shadowing bilateral testicular masses (white arrows).

**Figure 2:**
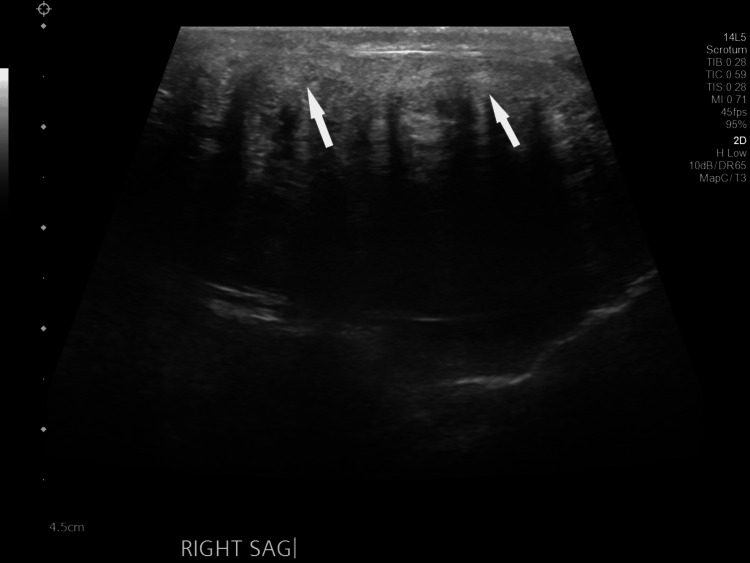
Grey-scale sagittal ultrasound image of the right testicle demonstrating shadowing masses consistent with testicular adrenal rest tumors in this patient with a history of congenital adrenal hyperplasia (white arrows).

Testicular Doppler ultrasound revealed multiple, echogenic lesions with areas of acoustic shadowing in both testes (Figure 1 and Figure 2). A testicular ultrasound report from an outside hospital seven years prior described multiple bilateral testicular masses. Lab values were consistent with CAH: in a recent test, 17-hydroxyprogesterone was severely elevated, 18420.75 ng/dL (reference value < 138.00 ng/dL). ACTH was also elevated, 1478.0 pg/mL (reference value 7.2-63.3 pg/mL). Serum dehydroepiandrosterone sulfate (DHEA-S) was decreased, 74 ug/dL (reference value 80-560 ug/dL). Other pertinent lab values such as lactate dehydrogenase (LDH), beta human chorionic gonadotropin (β-hCG), alpha-fetoprotein (AFP), and urinalysis were unremarkable. Considering the benign nature of TARTs, urology recommended medical management for CAH to prevent further tumor enlargement. No surgical intervention was indicated as the patient was asymptomatic. The patient declined a spermogram evaluation citing that he had no desire of having children. Thus, no further diagnostic workup for infertility was pursued. 

## Discussion

The most common cause of CAH (> 90%) is 21-hydroxylase deficiency [2]. The severity of CAH exists on a spectrum due to the variability in the amount of residual enzyme activity. With aldosterone and cortisol deficiencies, patients often present with hypotension, hypoglycemia, and hyperpigmentation. CAH occasionally causes life-threatening adrenal crisis. TART is linked to poor hormonal control in patients with CAH [4]. It has been hypothesized that chronically elevated ACTH stimulates hyperplasia of pluripotent cells in the testes to become TARTs, as ACTH receptors are present in TART tissues [1].

CAH is commonly discovered on newborn screening. A diagnosis of CAH can be established when the random 17-hydroxyprogesterone level is above 1000 ng/dL, although most affected infants have levels well above 5000 ng/dL [5]. When a diagnosis of classical CAH (21-hydroxylase deficiency) is highly probable, additional workup such as ACTH stimulation tests, genetic testing, and adrenal ultrasound is not necessary. In this case, the significantly elevated 17-hydroxyprogesterone and ACTH levels are highly suggestive of CAH. Alternative diagnoses such as germ cell tumors or infectious etiologies are not as likely since the serum markers suggestive of germ cell tumors and urinalysis are normal.

Ultrasound is the modality of choice in the evaluation of TARTs. TARTs are often bilateral, well-defined, hypoechoic masses located within the mediastinum testis on sonography [6]. In our case, the testicular ultrasound appearance is atypical. Rather than hypoechoic, the testicular parenchyma is diffusely heterogeneous with shadowing areas of increased echogenicity. It has been shown that the presentation of TARTs on ultrasound can be variable; most (83%) are hypoechogenic, but in rare cases (6%) they may be hyperechogenic [1]. Metastatic disease is unlikely in our patient as the bilateral testicular lesions have been present for over seven years. It remains a diagnostic challenge to distinguish TARTs from Leydig cell tumors (LCTs) given their similarity in morphology. However, specific patient characteristics point to a diagnosis of TARTs: a history of CAH, presence for over seven years, young age, history of poor therapeutic adherence, and a lack of evidence suggestive of metastasis [7]. Most importantly, TARTs are present in 37% of patients with CAH whereas LCTs in CAH have rarely been described [7]. Given the above reasons, we believe that our case is an unusual presentation of TARTs rather than other types of testicular tumors or infection.

Currently, limited treatment options exist for TARTs. Current therapy focuses on the treatment of underlying CAH and the preservation of fertility. Medical therapy involves aggressive steroid replacement therapy to suppress ACTH with the hopes of shrinking the tumor [8]. Surgery has not been shown to restore fertility [9]. Thus, it is indicated only in patients with severe pain.

In our patient with CAH, the TARTs likely arose as a result of poor hormonal control from nonadherence with glucocorticoid replacement therapy. Timely diagnosis is critical because, if left unmanaged, TARTs may cause impairment of testicular function leading to irreversible infertility. In a case series of six patients with CAH and TARTs, five had abnormal semen analysis [10]. Clinicians should have a high index of suspicion for TARTs in male patients with CAH and offer routine screening with ultrasound. Semen cryopreservation can be offered promptly to those at high risk of developing infertility.

## Conclusions

In this report, we detail a case of TART in a patient with poorly controlled CAH. Clinicians should maintain a high suspicion for TARTs in patients with CAH and not mistake these lesions for malignancy or infection to prevent unnecessary surgery or antibiotic treatment and a delay in initiating steroid replacement therapy. Medication adherence can help prevent the development of TARTs. The prompt diagnosis of TARTs in men with CAH can allow them to take measures to preserve fertility prior to deterioration of testicular function.
